# Supplementary material for: Iterative improvement in the automatic modular design of robot swarms
Source: PeerJ Comput Sci. 2020 Dec 7;6:e322. doi: 10.7717/peerj-cs.322 (PMC7924708; doi:10.7717/peerj-cs.322)
Supplement: Supplemental Information 3 [file peerj-cs-06-322-s003.zip › argos3/doc/api/standalone/a00512_source.html]

ARGoS: plugins/robots/generic/control\_interface/ci\_quadrotor\_speed\_actuator.cpp Source File


- Main Page
- Related Pages
- Namespaces
- Classes
- Files

- File List
- File Members

# plugins/robots/generic/control\_interface/ci\_quadrotor\_speed\_actuator.cpp

Go to the documentation of this file.

```
00001 
00007 #include "ci_quadrotor_speed_actuator.h"
00008 
00009 #ifdef ARGOS_WITH_LUA
00010 #include <argos3/core/wrappers/lua/lua_utility.h>
00011 #endif
00012 
00013 namespace argos {
00014 
00015    /****************************************/
00016    /****************************************/
00017 
00018 #ifdef ARGOS_WITH_LUA
00019    /*
00020     * The stack must have three values in this order:
00021     * 1. x coordinate of the velocity (a number)
00022     * 2. y coordinate of the velocity (a number)
00023     * 3. z coordinate of the velocity (a number)
00024     */
00025    int LuaSetQuadRotorLinearVelocity(lua_State* pt_lua_state) {
00026       /* Check parameters */
00027       if(lua_gettop(pt_lua_state) != 3) {
00028          return luaL_error(pt_lua_state, "robot.quadrotor.set_linear_velocity() expects 3 arguments");
00029       }
00030       luaL_checktype(pt_lua_state, 1, LUA_TNUMBER);
00031       luaL_checktype(pt_lua_state, 2, LUA_TNUMBER);
00032       luaL_checktype(pt_lua_state, 3, LUA_TNUMBER);
00033       /* Perform action */
00034       CLuaUtility::GetDeviceInstance<CCI_QuadRotorSpeedActuator>(pt_lua_state, "quadrotor")->
00035          SetLinearVelocity(CVector3(lua_tonumber(pt_lua_state, 1),
00036                                     lua_tonumber(pt_lua_state, 2),
00037                                     lua_tonumber(pt_lua_state, 3)));
00038       return 0;
00039    }
00040 #endif
00041 
00042    /****************************************/
00043    /****************************************/
00044 
00045 #ifdef ARGOS_WITH_LUA
00046    /*
00047     * The stack must have one value:
00048     * 1. x coordinate of the velocity (a number)
00049     */
00050    int LuaSetQuadRotorRotationalSpeed(lua_State* pt_lua_state) {
00051       /* Check parameters */
00052       if(lua_gettop(pt_lua_state) != 1) {
00053          return luaL_error(pt_lua_state, "robot.quadrotor.set_rotational_speed() expects 1 argument");
00054       }
00055       luaL_checktype(pt_lua_state, 1, LUA_TNUMBER);
00056       /* Perform action */
00057       CLuaUtility::GetDeviceInstance<CCI_QuadRotorSpeedActuator>(pt_lua_state, "quadrotor")->
00058          SetRotationalSpeed(CRadians(lua_tonumber(pt_lua_state, 1)));
00059       return 0;
00060    }
00061 #endif
00062 
00063    /****************************************/
00064    /****************************************/
00065 
00066 #ifdef ARGOS_WITH_LUA
00067    void CCI_QuadRotorSpeedActuator::CreateLuaState(lua_State* pt_lua_state) {
00068       CLuaUtility::OpenRobotStateTable(pt_lua_state, "quadrotor");
00069       CLuaUtility::AddToTable(pt_lua_state, "_instance", this);
00070       CLuaUtility::AddToTable(pt_lua_state, "set_linear_velocity", &LuaSetQuadRotorLinearVelocity);
00071       CLuaUtility::AddToTable(pt_lua_state, "set_rotational_speed", &LuaSetQuadRotorRotationalSpeed);
00072       CLuaUtility::CloseRobotStateTable(pt_lua_state);
00073    }
00074 #endif
00075 
00076    /****************************************/
00077    /****************************************/
00078 
00079 }
```

---

Generated on 10 Jul 2018 for ARGoS by 
 1.6.1
